# Treatment With Vasopressor Agents for Cardiovascular Shock Patients With Poor Renal Function; Results From the Japanese Circulation Society Cardiovascular Shock Registry

**DOI:** 10.3389/fmed.2021.648824

**Published:** 2021-05-03

**Authors:** Tsukasa Yagi, Ken Nagao, Eizo Tachibana, Naohiro Yonemoto, Kazuo Sakamoto, Yasushi Ueki, Hiroshi Imamura, Takamichi Miyamoto, Hiroshi Takahashi, Hiroyuki Hanada, Nobutaka Chiba, Shigemasa Tani, Naoya Matsumoto, Yasuo Okumura

**Affiliations:** ^1^Japanese Circulation Society (JCS) Shock Registry Scientific Committee, Tokyo, Japan; ^2^Department of Cardiology, Kawaguchi Municipal Medical Center, Kawaguchi, Japan; ^3^Department of Cardiology, Nihon University Hospital, Tokyo, Japan; ^4^Department of Biostatistics, Kyoto University School of Public Health, Kyoto, Japan; ^5^Department of Cardiology, Saiseikai Fukuoka General Hospital, Fukuoka, Japan; ^6^Department of Emergency and Critical Care Center, Shinshu University School of Medicine, Matsumoto, Japan; ^7^Department of Cardiology, Musashino Red Cross Hospital, Musashino, Japan; ^8^Department of Cardiology, Steel Memorial Muroran Hospital, Muroran, Japan; ^9^Department of Emergency and Critical Care Center, Aomori Prefectural Central Hospital, Aomori, Japan; ^10^Department of Emergency and Critical Care Medicine, Nihon University Hospital, Tokyo, Japan; ^11^Division of Cardiology, Department of Medicine, Nihon University School of Medicine, Tokyo, Japan

**Keywords:** cardiogenic shock, cardiovascular disease, vasopressor agents, norepinephrine, dopamine

## Abstract

According to the guidelines for cardiogenic shock, norepinephrine is associated with fewer arrhythmias than dopamine and may be the better first-line vasopressor agent. This study aimed to evaluate the utility of norepinephrine vs. dopamine as first-line vasopressor agent for cardiovascular shock depending on the presence and severity of renal dysfunction at hospitalization. This was a secondary analysis of the prospective, multicenter Japanese Circulation Society Cardiovascular Shock Registry (JCS Shock Registry) conducted between 2012 and 2014, which included patients with shock complicating emergency cardiovascular disease at hospital arrival. The analysis included 240 adult patients treated with norepinephrine alone (*n* = 98) or dopamine alone (*n* = 142) as the first-line vasopressor agent. Primary endpoint was mortality at 30 days after hospital arrival. The two groups had similar baseline characteristics, including estimated glomerular filtration rate (eGFR), and similar 30-day mortality rates. The analysis of the relationship between 30-day mortality rate after hospital arrival and vasopressor agent used in patients categorized according to the eGFR-based chronic kidney disease classification revealed that norepinephrine as the first-line vasopressor agent might be associated with better prognosis of cardiovascular shock in patients with mildly compromised renal function at admission (0.0 vs. 22.6%; *P* = 0.010) and that dopamine as the first-line vasopressor agent might be beneficial for cardiovascular shock in patients with severely compromised renal function [odds ratio; 0.22 (95% confidence interval 0.05–0.88; *P* = 0.032)]. Choice of first-line vasopressor agent should be based on renal function at hospital arrival for patients in cardiovascular shock.

**Clinical Trial Registration:** http://www.umin.ac.jp/ctr/, Unique identifier: 000008441.

## Introduction

The number of patients with chronic kidney disease (CKD) has been markedly increasing worldwide (1). CKD is an important risk factor for cardiovascular events and accounts for all-cause mortality in patients with cardiovascular disease (CVD) (2–5). Moreover, cardiogenic shock is a serious cardiovascular event associated with a high mortality rate (6–8). In patients with cardiogenic shock, vasopressor agents are indicated in patients with severe or persistent hypotension despite fluid administration, and various vasopressor agents have been used for the treatment of cardiogenic shock. According to the clinical statements and guidelines for the management of cardiogenic shock, norepinephrine is associated with fewer arrhythmias and is therefore the vasopressor agent of choice in many patients with cardiogenic shock; however, the optimal first-line vasopressor agent for cardiogenic shock patients remains unclear (9, 10). To our knowledge, no clinical studies to date have investigated the effects of first-line vasopressor agents for cardiogenic shock in patients with poor renal function.

We previously reported that estimated glomerular filtration rate (eGFR) was a strong predictor of 30-day mortality in patients in cardiovascular shock (11); consistent with this finding, other studies also reported that a history of CKD, presence of renal dysfunction at hospitalization, and acute renal dysfunction were strong predictors of 30-day mortality in patients in cardiogenic shock (12–15). In addition, some vasopressor agents are known to impact renal blood flow. Specifically, the administration of norepinephrine usually results in reduced blood flow to organs, particularly to kidneys (16), and the administration of low-dose dopamine, which selectively activates dopamine-specific receptors in renal and visceral blood vessels, results in increased blood flow to kidneys (17).

Therefore, we aimed to evaluate the utility of norepinephrine vs. dopamine for patients in cardiovascular shock and to elucidate the efficacy of vasopressors based on the presence and extent of renal dysfunction at the time of hospitalization in these patients.

## Methods

### Study Design

This was a secondary analysis of the Japanese Circulation Society Cardiovascular Shock Registry (JCS Shock Registry) (11). We have previously conducted a prospective, observational, multicenter cohort study based on the JCS Shock Registry (11, 18, 19). Patients diagnosed with cardiovascular shock complicating emergency CVD were registered from 82 centers in Japan between May 2012 and June 2014 (11). Maintenance of the registry was approved by the ethics committee of each participant hospital, and the present study was registered with the University Hospital Medical Information Network Clinical Trials Registry (UMIN000008441; http://www.umin.ac.jp/ctr/index/htm/). We prepared the manuscript according to the strengthening the reporting of observational studies in epidemiology (STROBE) guidelines.

The design and data collection methods for the JCS Shock Registry have been reported previously (11). Briefly, patients eligible for inclusion in the JCS Shock Registry had out-of-hospital onset of cardiovascular shock and met one major criterion and one or more minor criteria described below. The major criteria were systolic blood pressure ≤ 100 mmHg, including decline of systolic blood pressure by >30 mmHg from the usual value, and heart rate < 60 beats/min or > 100 beats/min. The minor criteria were the presence of cold sweating, skin pallor, cyanosis, capillary refill time > 2 s, and altered consciousness. Patients who had out-of-hospital cardiac arrest without return of spontaneous circulation on arrival at hospital and those younger than 16 years of age were excluded. The causes of cardiovascular shock included acute coronary syndrome, non-ischemic arrhythmia, aortic disease, myocarditis, cardiomyopathy, pulmonary thromboembolism, valvular heart disease, infective endocarditis, cardiac tamponade, and others.

### Study Patients

Patients from the JCS Shock Registry who were administered intravenous norepinephrine alone or dopamine alone as the first vasopressor agent within 24 h of arrival at the emergency department (ED) were included in the present study. Patients who received both agents were excluded because it was difficult to determine which of the two drugs was used initially or whether both were used simultaneously. In addition, patients who received intra-aortic balloon pump (IABP) support and/or veno-arterial extracorporeal membrane oxygenation (VA ECMO) were excluded for the same reason.

To analyze the relationship between mortality rate at 30 days after hospital arrival and vasopressor agent use, the patients were categorized into four groups according to the eGFR-based CKD classification: CKD stage G0/1/2, eGFR > 60 mL/min/1.73 m^2^ (Group G0/1/2); CKD stage G3a, eGFR 45–59 mL/min/1.73 m^2^ (Group G3a); CKD stage G3b, eGFR 30–44 mL/min/1.73 m^2^ (Group G3b); and CKD stage G4/5, eGFR <30 mL/min/1.73 m^2^ (Group G4/5). The following three-variable Japanese equation for GFR estimation based on serum creatinine (SCr) level and age, which is recommended in both clinical settings and epidemiological studies, was used: eGFR = 194 × SCr^−1.094^ × age^−0.287^ × 0.739 (if female) (20).

### Endpoints

The primary endpoint was mortality rate at 30 days after hospital arrival in the entire study population. The secondary endpoint was the 30-day mortality rate after hospital arrival in each group.

### Statistical Methods

Data were expressed as medians with interquartile range for continuous variables and as percentages for discrete variables. Baseline characteristics of the subjects enrolled in the present study were compared using the chi-square test for categorical variables and the Mann-Whitney *U* test for continuous variables, as appropriate. The primary endpoint was compared using the chi-square test, and a *P* ≤ 0.05 were considered to indicate statistical significance.

Trends were examined using univariate regression models. Multivariable logistic regression analysis was used to assess the contribution of the administered vasopressor agent to 30-day mortality after hospital arrival, and odds ratios (ORs) with 95% confidence intervals (CIs) were calculated. Potential confounding factors based on biological plausibility and previous studies were included in the multivariable logistic regression analysis. These variables included age, sex (male, female), systolic blood pressure at ED arrival, heart rate at ED arrival, respiratory rate at ED arrival, pathophysiology of shock (pump, non-pump, including volume, and rate), CKD stage based on eGFR, and administration of vasopressor agents (norepinephrine or dopamine) (6, 11, 21).

## Results

### Patient Population

**Figure 1:**
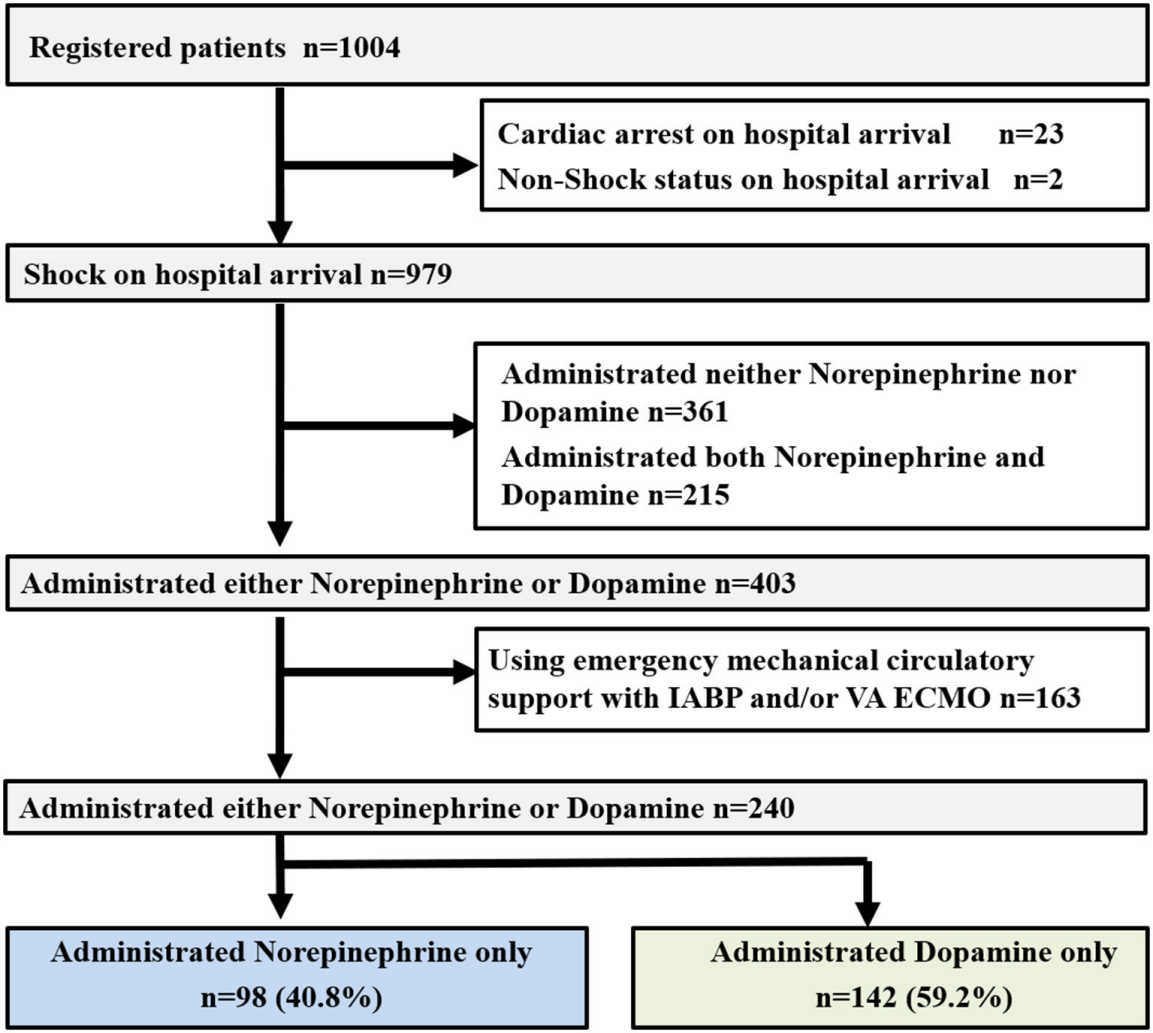
Study profile. OHCA, out-of-hospital cardiac arrest; ROSC, return of spontaneous circulation; IABP, intra-aortic balloon pumping; VA ECMO, veno-arterial extracorporeal membrane oxygenation.

Of a total of 1,004 patients entered in the JCS Shock Registry, 979 eligible patients were included in the present study (Figure 1) (11). Among these, 361 patients did not receive norepinephrine or dopamine and 215 patients received both norepinephrine and dopamine. Among the remaining 403 patients who received norepinephrine or dopamine alone, 163 patients who received IABP and/or VA ECMO support were excluded. Therefore, the final study population included the remaining 240 patients (Figure 1).

### Baseline Characteristics

In the study population of 240 patients, 98 (40.8%) and 142 (59.2%) patients received norepinephrine alone and dopamine alone, respectively, and the number of patients administered dopamine alone was significantly higher than that of patients administered norepinephrine alone (*P* = 0.005). The characteristics of the patients are presented in Table 1. There were no significant differences in the baseline characteristics between the norepinephrine and dopamine groups. According to the eGFR-based CKD classification, the study population included 46, 57, 72, and 62 patients in Group G0/1/2, G3a, G3b, and G4/5, respectively.

**Table 1:** Patient characteristics.

**Characters**	**Norepinephrine group (*n =* 98)**	**Dopamine group (*n =* 142)**	***P-*value**
Age-yr median (IQR)	75 (67–82)	76 (67–83)	0.977
Male sex—no. (%)	60 (61.2)	83 (58.5)	0.668
The pathophysiology of shock—no. (%)			0.387
Pump	57 (58.2)	70 (49.3)	
Volume	20 (20.4)	37 (26.1)	
Rate	21 (21.4)	35 (24.6)	
Cardiogenic source—no. (%)			0.261
ACS	45 (45.9)	46 (32.4)	
Arrhythmia	10 (10.2)	28 (19.7)	
Aortic disease	15 (15.3)	44 (31.0)	
The others	28 (28.6)	24 (16.9)	
SBP on ED arrival-mmHg median (IQR)	72 (50–85)	78 (60–87)	0.100
HR on ED arrival—beats/min median (IQR)	67 (39–98)	72 (42–102)	0.430
RR on ED arrival—per min median (IQR)	20 (15–25)	20 (12–26)	0.971
BT on ED—degree centigrade median (IQR)	36.0 (35.0–36.0)	36.0 (35.0–36.0)	0.586
Arterial pH on ED arrival^*^ median (IQR)	7.32 (7.14–7.39)	7.30 (7.18–7.38)	0.904
Arterial Lactate on ED arrival—mmol/l^**^ median (IQR)	2.90 (1.00–7.50)	4.90 (2.80–7.80)	0.379
eGFR at ED arrival^§^ median (IQR)	43.7 (27.1–55.4)	43.1 (30.1–59.6)	0.255
LVEF on ED arrival median (IQR)	49.0 (35.5–60.0)	50.0 (39.0–65.5)	0.191
Heart failure,—no. (%)	57 (58.8)	78 (55.7)	0.641
Out-of–hospital cardiac arrest before hospital arrival,—no. (%)	24 (24.5)	38 (26.8)	0.693
Mechanical ventilation—no. (%)	38 (38.8)	63 (44.4)	0.389
Continuous hemodiafiltration—no. (%)	2 (2.0)	4 (2.8)	0.698
Patients treated anti-arrhythmic agents within 12 h of arrival at ED—no. (%)	7 (7.1)	12 (8.5)	0.083
The volume of infusion within 30 min of arrival at ED—no. (%)^#^			0.141
≤ 500 ml	8/20 (40.0)	8/37 (21.6)	
> 500 ml	12/20 (60.0)	29/37 (78.4)	

* *the arterial ph was recorded for 72 patients in the norepinephrine group and for 115 patients in the dopamine group*.

** *the arterial lactate was recorded for 55 patients in the norepinephrine group and for 83 patients the dopamine group*.

§ *the estimated glomerular filtration rate (eGFR) was recorded for 97 patients in the norepinephrine group and for 140 patients in the dopamine group*.

# *the number of patients who treated the volume of infusion within 30 min of arrival at emergency department was divided by the number of patients who the pathophysiology of shock was “volume”*.

*ACS, acute coronary syndrome; SBP, systolic blood pressure; ED, emergency department; IQR, interquartile range; HR, heart rate; RR, respiratory rate; BT, body temperature; LVEF, left ventricular ejection fraction*.

### Primary Outcome

Table 2 shows the outcomes in the entire study population. The mortality rate at 30-day after hospital arrival was 26.5% in the norepinephrine group and 25.4% in the dopamine group (*P* = 0.838). In the multivariate logistic regression analysis including the entire study population, the adjusted OR for 30-day mortality in the dopamine group compared to the norepinephrine group was 1.00 (95%CI 0.48–2.09; *P* = 0.994) (Table 2). In addition, the 30-day mortality rate was lower in the subgroups with better renal function (Group G0/1/2 vs. G3a, G3b, and G4/5 13.0 vs. 12.3, 33.3, and 38.7%; *P* = 0.001). In the multivariate logistic regression analysis of the entire study population, the adjusted ORs for 30-day mortality in the patients with Group G3a, G3b, and G4/5 were 0.97 (95%CI 0.25–3.66; *P* = 0.959), 3.33 (95%CI 1.24–8.95; *P* = 0.135), and 4.21 (95%CI 1.55–12.2; *P* = 0.029), respectively, compared to the patients with Group G0/1/2 (Table 2). Similar results were observed based on the analysis of the norepinephrine group alone (Supplementary Table 1). Conversely, in the dopamine group (Supplementary Table 2), no significant differences in 30-day mortality rate were noted among the four groups.

**Table 2:** In entire study population, factors associating with 30-day mortality after hospital arrival.

**Variable**	**Mortality (%)**	**Crude OR (95%CI)**	**Adjusted OR (95%CI)**	***P-*value**
Age—yr		1.029 (1.002–1.057)	1.037 (0.998–1.078)	0.065
Sex				
Female	25/97 (25.8)	(Reference)	(Reference)	
Male	37/143 (25.9)	1.005 (0.558–1.812)	1.341 (0.617–2.916)	0.459
SBP on ED arrival		0.990 (0.980–0.999)	0.990 (0.977–1.004)	0.164
HR on ED arrival		1.001 (0.994–1.009)	1.003 (0.993–1.013)	0.570
RR on ED arrival		0.968 (0.937–0.999)	0.979 (0.940–1.020)	0.318
The pathophysiology of shock^*^				
Non—pump	22/113 (19.5)	(Reference)	(Reference)	
Pump	40/127 (31.5)	1.902 (1.046–3.457)	2.278 (1.062–4.888)	0.035
eGFR
G0/1/2	6/46 (13.0)	(Reference)	(Reference)	
G3a	7/57 (12.3)	0.933 (0.291–2.998)	0.966 (0.255–3.657)	0.959
G3b	24/72 (33.3)	3.333 (1.241–8.954)	2.463 (0.756–8.032)	0.135
G4/5	24/62 (38.7)	4.211 (1.551–11.43)	3.748 (1.148–12.24)	0.029
Administration of vasopressor agents				
Norepinephrine	26/98 (26.5)	(Reference)	(Reference)	
Dopamine	36/142 (25.4)	0.940 (0.523–1.691)	1.003 (0.480–2.094)	0.994

* *the pathophysiology of shock consisted of pump and non-pump including volume and rate. OR, odds ratio; CI, confidence interval; SBP, systolic blood pressure; ED, emergency department; HR, heart rate; eGFR, estimated glomerular filtration rate*.

### Secondary Outcome

We examined the relationship between the 30-day mortality rate and vasopressor use in each group according to the CKD stage (Table 3). The characteristics of the patients in each group are presented in Supplementary Tables 3–6. Among the patients with stage Group G3a, the 30-day mortality rate was significantly lower in the norepinephrine group than in the dopamine group (0.0 vs. 22.6%; *P* = 0.010). However, among the patients with Group G4/5, the mortality rate tended to be higher in the norepinephrine group than in the dopamine group (51.9 vs. 28.6%; *P* = 0.062). Furthermore, there was no significant difference in the 30-day mortality rate between the norepinephrine vs. dopamine groups among patients with Group G0/1/2 (norepinephrine vs. dopamine, 13.3 vs. 12.9%; *P* = 0.968) and among those with Group G3b (norepinephrine vs. dopamine, 34.5 vs. 32.6%; *P* = 0.865). In the multivariate logistic regression analysis, the adjusted ORs for 30-day mortality in the dopamine group compared to the norepinephrine group were 15.1 (95%CI 0.08–2,928, *P* = 0.312), 0.60 (95%CI 0.15–2.47; *P* = 0.474), and 0.22 (95%CI 0.05–0.88; *P* = 0.032) in patients with Group G0/1/2, G3b, and G4/5, respectively.

**Table 3:** Relation between administration of vasopressor agents and 30-day Mortality After Hospital Arrival in each group.

**eGFR (mL/min/** **1.73 m^**2**^)**	**Variable**	**Mortality (%)**	**Crude OR (95%CI)**	**Adjusted OR (95%CI)**	***P-*value**
> 60 (G0/1/2)	Norepinephrine	2/15 (13.3)	(Reference)	(Reference)	
	Dopamine	4/31 (12.9)	0.963 (0.156–5.954)	15.12 (0.078–2,928)	0.312
45–59 (G3a)	Norepinephrine	0/26 (0.0)	(Reference)	(Reference)	
	Dopamine	7/31 (22.6)	–	–	–
30–44 (G3b)	Norepinephrine	10/29 (34.5)	(Reference)	(Reference)	
	Dopamine	14/43 (32.6)	0.917 (0.339–2.485)	0.598 (0.145–2.466)	0.477
< 30 (G4/5)	Norepinephrine	14/27 (51.9)	(Reference)	(Reference)	
	Dopamine	10/35 (28.6)	0.371 (0.130–1.064)	0.217 (0.054–0.877)	0.032

*eGFR, estimated glomerular filtration rate; OR, odds ratio; CI, confidence interval*.

## Discussion

In the present study based on the largest nationwide registry of patients with cardiovascular shock caused by various causes of CVD, we assessed the actual use and utility of vasopressor agents for cardiovascular shock within 24 h of arrival at ED in patients with poor renal function. We showed the first-line vasopressor agent, such as norepinephrine and dopamine, should be chosen based on renal function at hospital arrival for patients in cardiovascular shock. As the patients in the present study reflect the real-world situation, our findings have important implications for clinical practice, especially in cases where determining the origin of cardiovascular shock is difficult at hospital arrival.

The present study indicated that the 30-day mortality rate was significantly lower in patients with eGFR 45–59 ml/min/1.73 m^2^ treated with norepinephrine compared to those treated with dopamine. Conversely, among patients with eGFR < 30 ml/min/1.73 m^2^, the 30-day mortality rate was significantly higher in those treated with norepinephrine than in those treated with dopamine. In a randomized multicenter study (6), the 28-day mortality rate for patients in shock did not differ significantly between those administered dopamine and those administered norepinephrine as the vasopressor agent of first choice, although the incidence of arrhythmias was higher in patients treated with dopamine than in those treated with norepinephrine as the initial vasopressor agent. In addition, the same study reported that the dopamine treatment was more closely associated with increased 28-day mortality rate than the norepinephrine treatment for patients in cardiogenic shock whereas a similar association was not found for patients in septic or hypovolemic shock (6). Some studies have also recommended norepinephrine as the vasopressor agent of first choice as it primarily stimulates alpha adrenergic receptors, causing an elevation in systemic vascular resistance in a volume-dependent manner, and modestly stimulates cardiac beta adrenergic receptors, thereby aiding in the maintenance of cardiac output (10, 22). Thus, norepinephrine may be superior to dopamine, considering the lower risk of adverse reactions including tachycardia and other arrhythmias, which is the presumed cause for its recommendation for patients in shock (10, 22). However, they did not comment on the utility of norepinephrine or dopamine in patients with poor renal function.

The present study revealed that the 30-day mortality rate was significantly higher in patients with an eGFR <45 mL/min/1.73 m^2^ than in those with an eGFR > 45 mL/min/1.73 m^2^ among those treated with norepinephrine. However, the 30-day mortality rate in patients treated with dopamine as the initial vasopressor agent was similar regardless of the level of renal function at admission. Numerous epidemiological studies have demonstrated that the progression of nephropathy is directly associated with an increase in the frequency of cardiovascular events (3, 23–25). An observational study involving 1.12 million adults in the United States has revealed that the mortality rate and CVD incidence increased significantly with decreasing eGFR (3). The rate of increase in CVD-associated mortality rate is higher than the rate of decline in renal function, indicating the importance of suppressing the risk of death from CVD in addition to suppressing CKD progression as a major treatment goal in patients with CKD (26, 27). A major reason for this proposal was the finding that an increase in CKD stage from G3a (eGFR 45–59 mL/min/1.73 m^2^) to G3b (eGFR 30–44 mL/min/1.73 m^2^) was associated with a marked increase in the risk for CVD and the onset of terminal renal failure (28).

The present study has several limitations. First, this was not a randomized controlled trial. Second, details of the treatment, such as administration of norepinephrine and/or dopamine and administration of the other agents, were left to the physician's discretion at each hospital. The exact norepinephrine and dopamine doses and durations used in the study patients were not always known. Dopamine is an endogenous catecholamine and serves as a neurotransmitter and norepinephrine precursor. Dopamine activates diverse receptors in a dose-dependent manner. Specifically, low-dose dopamine selectively activates dopamine receptors of the renal and visceral blood vessels, leading to increased blood flow (17). Low-dose dopamine also acts directly on tubular epithelial cells, causing an increase in the excretion of sodium into urine, which is not dependent on the increase of renal blood flow (29). Conversely, norepinephrine stimulates alpha adrenergic receptors, causing a blood volume-dependent increase in systemic vascular resistance. This vasoconstrictive activity usually leads to reduced organ blood flow, in particular renal blood flow (16). Therefore, the optimal doses and durations of these vasopressor agents should be elucidated in future studies. Third, the present study excluded patients who received IABP or VA ECMO support; the analyses including these patients did not reveal a significant difference in the 30-day mortality rate after hospital arrival between those treated with dopamine and those treated with norepinephrine as the initial vasopressor agent. Furthermore, in both patients with and without compromised renal function at admission, the multivariate logistic regression analysis revealed a minimal difference in the 30-day mortality between patients treated with norepinephrine and dopamine as the initial vasopressor agent. Fourth, details of the treatment, including to decide vasopressor agents, were left to the physician's discretion at each hospital. Finally, SCr and not urine output was recorded at admission in the study population, and it was often difficult to confirm whether the patients had acute kidney injury or CKD. Among 979 patients in the JCS Shock Registry, the median time from onset to hospital arrival was 72 min (interquartile range, 40–284 min) (11). Therefore, we considered that the eGFR values calculated at the time of ED arrival reflected the chronic renal function status. However, for severe shock patients, it might be important to decide vasopressor agents by eGFR at admission in ED despite acute kidney injury or CKD. In future studies, it is necessary to record eGFR and urine output overtime to clarify this issue.

In conclusion, the first-line vasopressor agent should be chosen based on renal function at hospital arrival for patients in cardiovascular shock. Furthermore, in patients with mildly compromised renal function at admission (eGFR 45–59 mL/min/1.73 m^2^), norepinephrine as the vasopressor agent of first choice might be associated with better prognosis. Conversely, in patients with severely compromised renal function (eGFR < 30 mL/min/1.73 m^2^), dopamine might be more beneficial as the first-line vasopressor agent. Future studies are warranted to elucidate optimal therapeutic strategies for patients with compromised renal function presenting with cardiovascular shock.

## Data Availability Statement

The original contributions presented in the study are included in the article/Supplementary Material, further inquiries can be directed to the corresponding author/s.

## Ethics Statement

The studies involving human participants were reviewed and approved by the University Hospital Medical Information Network Clinical Trials Registry (UMIN000008441; http://www.umin.ac.jp/ctr/index/htm/). The patients/participants provided their written informed consent to participate in this study.

## Author Contributions

TY, KN, ET, and NY analyzed data. TY, KN, KS, YU, HI, TM, HT, and HH interpreted data. TY, KN, ET, NC, and ST wrote the paper. TY, KN, ET, NM, and YO reviewed and edited the paper. TY, KN, ET, NY, KS, YU, HI, TM, HT, and HH designed the overall study. All authors contributed to the article and approved the submitted version.

## Conflict of Interest

The authors declare that the research was conducted in the absence of any commercial or financial relationships that could be construed as a potential conflict of interest.

## References

[1] GBD Chronic Kidney Disease Collaboration. Global, regional, and national burden of chronic kidney disease, 1990-2017: a systematic analysis for the Global Burden of Disease Study 2017. Lancet. (2020) 395:709–33. 10.1016/S0140-6736(20)30045-3 32061315 PMC7049905

[2] Anavekar NS McMurray JJ Velazquez EJ Solomon SD Kober L Rouleau JL. Relation between renal dysfunction and cardiovascular outcomes after myocardial infarction. N Engl J Med. (2004) 351:1285–95. 10.1056/NEJMoa041365 15385655

[3] Go AS Chertow GM Fan D McCulloch CE Hsu CY. Chronic kidney disease and the risks of death, cardiovascular events, and hospitalization. N Engl J Med. (2004) 351:1296–305. 10.1056/NEJMoa041031 15385656

[4] Niizuma S Nakamura S Ishibashi-Ueda H Yoshihara F Kawano Y. Kidney function and histological damage in autopsy subjects with myocardial infarction. Ren Fail. (2011) 33:847–52. 10.3109/0886022X.2011.605531 21823900

[5] Sarnak MJ Levey AS Schoolwerth AC Coresh J Culleton B Hamm LL. Kidney disease as a risk factor for development of cardiovascular disease: a statement from the American heart association councils on kidney in cardiovascular disease, high blood pressure research, clinical cardiology, and epidemiology and prevention. Hypertension. (2003) 42:1050–65. 10.1161/01.HYP.0000102971.85504.7c 14604997

[6] De Backer D Biston P Devriendt J Madl C Chochrad D Aldecoa C. Comparison of dopamine and norepinephrine in the treatment of shock. N Engl J Med. (2010) 362:779–89. 10.1056/NEJMoa0907118 20200382

[7] Varpula M Tallgren M Saukkonen K Voipio-Pulkki LM Pettila V. Hemodynamic variables related to outcome in septic shock. Intensive Care Med. (2005) 31:1066–71. 10.1007/s00134-005-2688-z 15973520

[8] Marchick MR Kline JA Jones AE. The significance of non-sustained hypotension in emergency department patients with sepsis. Intensive Care Med. (2009) 35:1261–4. 10.1007/s00134-009-1448-x 19238354 PMC2923927

[9] van Diepen S Katz JN Albert NM Henry TD Jacobs AK Kapur NK. Contemporary management of cardiogenic shock: a scientific statement from the American heart association. Circulation. (2017) 136:e232–68. 10.1161/CIR.0000000000000525 28923988

[10] Vincent JL De Backer D. Circulatory shock. N Engl J Med. (2013) 369:1726–34. 10.1056/NEJMra1208943 24171518

[11] Ueki Y Mohri M Matoba T Tsujita Y Yamasaki M Tachibana E. Characteristics and predictors of mortality in patients with cardiovascular shock in japan- results from the Japanese circulation society cardiovascular shock registry. Circ J. (2016) 80:852–9. 10.1253/circj.CJ-16-0125 27001192

[12] Kunadian V Qiu W Ludman P Redwood S Curzen N Stables R. Outcomes in patients with cardiogenic shock following percutaneous coronary intervention in the contemporary era: an analysis from the BCIS database (British Cardiovascular Intervention Society). JACC Cardiovasc Interv. (2014) 7:1374–85. 10.1016/j.jcin.2014.06.017 25523531

[13] Katz JN Stebbins AL Alexander JH Reynolds HR Pieper KS Ruzyllo W. Predictors of 30-day mortality in patients with refractory cardiogenic shock following acute myocardial infarction despite a patent infarct artery. Am Heart J. (2009) 158:680–7. 10.1016/j.ahj.2009.08.005 19781431

[14] Samadi A Le Feuvre C Allali Y Collet JP Barthelemy O Beygui F. Medium-term survival after primary angioplasty for myocardial infarction complicated by cardiogenic shock after the age of 75 years. Arch Cardiovasc Dis. (2008) 101:175–80. 10.1016/S1875-2136(08)71800-X 18477945

[15] Koreny M Karth GD Geppert A Neunteufl T Priglinger U Heinz G. Prognosis of patients who develop acute renal failure during the first 24 hours of cardiogenic shock after myocardial infarction. Am J Med. (2002) 112:115–9. 10.1016/S0002-9343(01)01070-1 11835949

[16] Beale RJ Hollenberg SM Vincent JL Parrillo JE. Vasopressor and inotropic support in septic shock: an evidence-based review. Crit Care Med. (2004) 32(Suppl. 11):S455–65. 10.1097/01.CCM.0000142909.86238.B1 15542956

[17] Bayram M De Luca L Massie MB Gheorghiade M. Reassessment of dobutamine, dopamine, and milrinone in the management of acute heart failure syndromes. Am J Cardiol. (2005) 96:47G–58G. 10.1016/j.amjcard.2005.07.021 16181823

[18] Ueki Y Mohri M Matoba T Kadokami T Suwa S Yagi T. Prognostic value of neurological status on hospital arrival for short-term outcome in patients with cardiovascular shock- sub-analysis of the Japanese circulation society cardiovascular shock registry. Circ J. (2019) 83:1247–53. 10.1253/circj.CJ-18-1323 30944275

[19] Sakamoto K Matoba T Mohri M Ueki Y Tsujita Y Yamasaki M. Clinical characteristics and prognostic factors in acute coronary syndrome patients complicated with cardiogenic shock in Japan: analysis from the Japanese circulation society cardiovascular shock registry. Heart Vessels. (2019) 34:1241–9. 10.1007/s00380-019-01354-9 30715570

[20] Matsuo S Imai E Horio M Yasuda Y Tomita K Nitta K. Revised equations for estimated GFR from serum creatinine in Japan. Am J Kidney Dis. (2009) 53:982–92. 10.1053/j.ajkd.2008.12.034 19339088

[21] Sleeper LA Reynolds HR White HD Webb JG Dzavik V Hochman JS. A severity scoring system for risk assessment of patients with cardiogenic shock: a report from the SHOCK trial and registry. Am Heart J. (2010) 160:443–50. 10.1016/j.ahj.2010.06.024 20826251 PMC4229030

[22] Moller MH Claudius C Junttila E Haney M Oscarsson-Tibblin A Haavind A. Scandinavian SSAI clinical practice guideline on choice of first-line vasopressor for patients with acute circulatory failure. Acta Anaesthesiol Scand. (2016) 60:1347–66. 10.1111/aas.12780 27576362 PMC5213738

[23] Kokubo Y Nakamura S Okamura T Yoshimasa Y Makino H Watanabe M. Relationship between blood pressure category and incidence of stroke and myocardial infarction in an urban Japanese population with and without chronic kidney disease: the Suita Study. Stroke. (2009) 40:2674–9. 10.1161/STROKEAHA.109.550707 19478215

[24] Nakano T Ninomiya T Sumiyoshi S Fujii H Doi Y Hirakata H. Association of kidney function with coronary atherosclerosis and calcification in autopsy samples from Japanese elders: the Hisayama study. Am J Kidney Dis. (2010) 55:21–30. 10.1053/j.ajkd.2009.06.034 19765871

[25] Ninomiya T Kiyohara Y Kubo M Tanizaki Y Doi Y Okubo K. Chronic kidney disease and cardiovascular disease in a general Japanese population: the hisayama study. Kidney Int. (2005) 68:228–36. 10.1111/j.1523-1755.2005.00397.x 15954912

[26] Keith DS Nichols GA Gullion CM Brown JB Smith DH. Longitudinal follow-up and outcomes among a population with chronic kidney disease in a large managed care organization. Arch Intern Med. (2004) 164:659–63. 10.1001/archinte.164.6.659 15037495

[27] Adler AI Stevens RJ Manley SE Bilous RW Cull CA Holman RR. Development and progression of nephropathy in type 2 diabetes: the United Kingdom prospective diabetes study (UKPDS 64). Kidney Int. (2003) 63:225–32. 10.1046/j.1523-1755.2003.00712.x 12472787

[28] Levey AS de Jong PE Coresh J El Nahas M Astor BC Matsushita K. The definition, classification, and prognosis of chronic kidney disease: a KDIGO controversies conference report. Kidney Int. (2011) 80:17–28. 10.1038/ki.2010.483 21150873

[29] Kellum JA Decker JM. Use of dopamine in acute renal failure: a meta-analysis. Crit Care Med. (2001) 29:1526–31. 10.1097/00003246-200108000-00005 11505120

