# Supplementary material for: Treatment With Vasopressor Agents for Cardiovascular Shock Patients With Poor Renal Function; Results From the Japanese Circulation Society Cardiovascular Shock Registry
Source: Front Med (Lausanne). 2021 May 3;8:648824. doi: 10.3389/fmed.2021.648824 (PMC8126606; doi:10.3389/fmed.2021.648824)
Supplement: Supplementary file 1 [file Table_1.docx]

Supplementary Material

# Supplementary Tables

Supplementary Table 1. In the Norepinephrine Group, Factors Associating with 30-day Mortality After Hospital Arrival

| **Variable** | **Mortality (%)** | **Crude OR (95%CI)** | **Adjusted OR (95%CI)** | **P value** |
| --- | --- | --- | --- | --- |
| **Age- yr** |  | **1.037 (0.994-1.082)** | **1.018 (0.952-1.088)** | **0.232** |
| **Sex** |  |  |  |  |
| **Female** | **8/38 (21.1)** | **(Reference)** | **(Reference)** |  |
| **Male** | **18/60 (30.0)** | **1.607 (0.618-4.179)** | **3.425 (0.725-16.18)** | **0.120** |
| **SBP on ED arrival** |  | **0.990 (0.976-1.014)** | **0.968 (0.937-0.999)** | **0.045** |
| **HR on ED arrival** |  | **0.997 (0.986-1.009)** | **0.986 (0.965-1.008)** | **0.213** |
| **RR on ED arrival** |  | **0.985 (0.934-1.038)** | **1.029 (0.937-1.130)** | **0.556** |
| **The pathophysiology of shock*** |  |  |  |  |
| **Non- pump** | **10/41 (24.4)** | **(Reference)** | **(Reference)** |  |
| **Pump** | **16/57 (28.1)** | **1.210 (0.483-3.028)** | **3.435 (0.727-16.23)** | **0.119** |
| **eGFR** |  |  |  |  |
| **G0/1/2** | **2/15 (7.7)** | **(Reference)** | **(Reference)** |  |
| **G3a** | **0/26 (0.0)** | - | - | - |
| **G3b** | **10/29 (38.5)** | **3.421 (0.641-18.25)** | **19.07 (1.252-290.6)** | **0.034** |
| **G4/5** | **14/27 (53.8)** | **7.000 (1.319-37.15)** | **51.69 (2.888-925.1)** | **0.007** |

*The pathophysiology of shock consisted of pump and non-pump including volume and rate. OR, odds ratio; CI, confidence interval; SBP, systolic blood pressure; HR, heart rate; eGFR, estimated glomerular filtration rate.

Supplementary Table 2. In the Dopamine Group, Factors Associating with 30-day Mortality After Hospital Arrival

| **Variable** | | **Mortality (%)** | **Crude OR (95%CI)** | | **Adjusted OR (95%CI)** | **P value** |  |  |
| --- | --- | --- | --- | --- | --- | --- | --- | --- |
| **Age- yr** | |  | **1.024** | | **1.052 (0.994-1.114)** | **0.082** |  |  |
| **Sex** | |  |  | |  |  |  |  |
| **Female** | | **17/59 (28.8)** | **(Reference)** | | **(Reference)** |  |  |  |
| **Male** | | **19/83 (22.9)** | **0.733** | | **0.730 (0.256-2.084)** | **0.730** |  |  |
| **SBP on ED arrival** | |  | **0.989** | | **0.984 (0.965-1.003)** | **0.090** |  |  |
| **HR on ED arrival** | |  | **1.004** | | **1.013 (0.999-1.028)** | **0.077** |  |  |
| **RR on ED arrival** | |  | **0.958 (0.920-0.997)** | | **0.952 (0.904-1.003)** | **0.062** |  |  |
| **The pathophysiology of shock*** |  | | |  | | |  |  |
| **Non- pump** | | **12/72 (16.7)** | **(Reference)** | | **(Reference)** |  |  |  |
| **Pump** | | **24/70 (34.3)** | **2.609** | | **2.617 (0.986-6.945)** | **0.053** |  |  |
| **eGFR** | |  |  | |  |  |  |  |
| **G0/1/2** | | **4/31 (11.4)** | **(Reference)** | | **(Reference)** |  |  |  |
| **G3a** | | **7/31 (20.0)** | **1.969 (0.512-7.563)** | | **1.682 (0.347-8.150)** | **0.518** |  |  |
| **G3b** | | **14/43 (40.0)** | **3.259 (0.954-11.13)** | | **1.326 (0.292-6.013)** | **0.715** |  |  |
| **G4/5** | | **10/35 (28.6)** | **2.700 (0.750-9.719)** | | **1.197 (0.255-5.606)** | **0.820** |  |  |

*The pathophysiology of shock consisted of pump and non-pump including volume and rate. OR, odds ratio; CI, confidence interval; SBP, systolic blood pressure; HR, heart rate; eGFR, estimated glomerular filtration rate.

Supplementary Table 3. Patient characteristics in Group G0/1/2

| **Characters** | **Norepinephrine group (n=15)** | **Dopamine group (n=31)** | **P value** |
| --- | --- | --- | --- |
| **Age-yr median (IQR)** | **71 (57– 82)** | **66 (52- 80)** | **0.565** |
| **Male sex - no. (%)** | **10 (66.7)** | **20 (64.5)** | **0.886** |
| **The pathophysiology of shock – no (%)** |  |  | **0.743** |
| **Pump** | **10 (66.7)** | **17 (54.8)** |  |
| **Volume** | **3 (20.0)** | **8 (25.8)** |  |
| **Rate** | **2 (13.3)** | **6 (19.4)** |  |
| **Cardiogenic source – no. (%)** |  |  | **0.141** |
| **ACS** | **8 (53.3)** | **12 (38.7)** |  |
| **Arrhythmia** | **3 (20.0)** | **7 (22.6)** |  |
| **Aortic disease** | **2 (13.3)** | **11 (35.5)** |  |
| **The others** | **2 (13.3)** | **1 (3.2)** |  |
| **SBP on ED arrival-mmHg median (IQR)** | **71 (- 78)** | **84 (72- 92)** | **0.022** |
| **HR on ED arrival- beats/min median (IQR)** | **66 (34- 102)** | **77 (53- 95)** | **0.590** |
| **RR on ED arrival- per min median (IQR)** | **18 (10- 25)** | **16 (12- 21)** | **0.686** |
| **BT on ED – degree centigrade median (IQR)** | **35.0 (34.5- 36.5)** | **36.0 (35.0- 36.0)** | **0.536** |
| **Arterial pH on ED arrival* median (IQR)** | **7.34 (7.12- 7.42)** | **7.31 (7.27- 7.35)** | **0.972** |
| **Arterial Lactate on ED arrival –mmol/l** median (IQR)** | **1.00 (0.40- 10.0)** | **4.20 (2.35- 7.90)** | **0.063** |
| **eGFR at ED arrival median (IQR)** | **72.4 (64.9 – 82.1)** | **67.0 (63.1 – 80.2)** | **0.391** |
| **LVEF on ED arrival median (IQR)** | **48.0 (30.5- 50.8)** | **56.5 (42.8- 65.5)** | **0.152** |
| **Heart failure, - no. (%)** | **7 (46.7)** | **14 (45.2)** | **0.923** |
| **Out-of–hospital cardiac arrest before hospital arrival, – no. (%)** | **6 (40.0)** | **9 (29.0)** | **0.457** |
| **Mechanical ventilation - no. (%)** | **8 (53.3)** | **13 (41.9)** | **0.467** |
| **Continuous hemodiafiltration - no. (%)** | **0 (0)** | **0 (0)** | - |
| **Patients treated anti-arrhythmic agents within 12 hours of arrival at ED- no. (%)** | **1 (6.7)** | **5 (16.1)** | **0.571** |
| **The volume of infusion within 30 minutes of arrival at ED – no. (%)^#^** |  |  | **0.011** |
| **≤ 500ml** | **2 / 3 (66.7)** | **0 / 8 (0)** |  |
| **> 500ml** | **1 / 3 (33.3)** | **8 / 8 (100)** |  |

*The arterial ph was recorded for 11 patients in the norepinephrine group and for 24 patients in the dopamine group.

**The arterial lactate was recorded for 9 patients in the norepinephrine group and for 21 patients the dopamine group.

#The number of patients who treated the volume of infusion within 30 minutes of arrival at emergency department was divided by the number of patients who the pathophysiology of shock was “volume”.

ACS, acute coronary syndrome; SBP, systolic blood pressure; ED, emergency department; IQR, interquartile range; HR, heart rate; RR, respiratory rate; BT, body temperature; LVEF, left ventricular ejection fraction.

Supplementary Table 4. Patient characteristics in Group G3a

| **Characters** | **Norepinephrine group (n=26)** | **Dopamine group (n=31)** | **P value** |
| --- | --- | --- | --- |
| **Age-yr median (IQR)** | **74 (65– 77)** | **75 (67- 82)** | **0.344** |
| **Male sex - no. (%)** | **18 (69.2)** | **18 (58.1)** | **0.384** |
| **The pathophysiology of shock – no (%)** |  |  | **0.238** |
| **Pump** | **17 (65.4)** | **14 (45.2)** |  |
| **Volume** | **5 (19.2)** | **12 (38.7)** |  |
| **Rate** | **4 (15.4)** | **5 (16.1)** |  |
| **Cardiogenic source – no. (%)** |  |  | **0.224** |
| **ACS** | **16 (61.5)** | **12 (38.7)** |  |
| **Arrhythmia** | **0 (0)** | **2 (6.5)** |  |
| **Aortic disease** | **5 (19.2)** | **12 (38.7)** |  |
| **The others** | **5 (19.2)** | **5 (16.1)** |  |
| **SBP on ED arrival-mmHg median (IQR)** | **69 (30- 80)** | **78 (62- 85)** | **0.071** |
| **HR on ED arrival- beats/min median (IQR)** | **42 (36- 84)** | **82 (45- 97)** | **0.027** |
| **RR on ED arrival- per min median (IQR)** | **17 (12- 22)** | **20 (17- 30)** | **0.144** |
| **BT on ED – degree centigrade median (IQR)** | **36.0 (35.0- 36.0)** | **36.0 (34.5- 36.0)** | **0.999** |
| **Arterial pH on ED arrival* median (IQR)** | **7.27 (7.04- 7.39)** | **7.29 (7.22- 7.36)** | **0.509** |
| **Arterial Lactate on ED arrival –mmol/l** median (IQR)** | **4.70 (1.30- 7.50)** | **5.55 (2.78- 8.13)** | **0.338** |
| **eGFR at ED arrival median (IQR)** | **53.3 (50.9 – 56.2)** | **52.5 (49.4 – 54.6)** | **0.543** |
| **LVEF on ED arrival median (IQR)** | **53.0 (40.0- 66.0)** | **50.0 (45.0- 61.0)** | **0.621** |
| **Heart failure, -no. (%)** | **15 (57.7)** | **12 (38.7)** | **0.153** |
| **Out-of–hospital cardiac arrest before hospital arrival, – no. (%)** | **6 (23.1)** | **7 (22.6)** | **0.965** |
| **Mechanical ventilation - no. (%)** | **17 (65.4)** | **17 (54.8)** | **0.419** |
| **Continuous hemodiafiltration - no. (%)** | **0 (0)** | **0 (0)** | - |
| **Patients treated anti-arrhythmic agents within 12 hours of arrival at ED- no. (%)** | **0 (0)** | **2 (6.5)** | - |
| **The volume of infusion within 30 minutes of arrival at ED – no. (%)^#^** |  |  | **0.949** |
| **≤ 500ml** | **2 / 5 (40.0)** | **5 / 12 (41.7)** |  |
| **> 500ml** | **3 / 5 (60.0)** | **7 / 12 (58.3)** |  |

*The arterial ph was recorded for 18 patients in the norepinephrine group and for 24 patients in the dopamine group.

**The arterial lactate was recorded for 15 patients in the norepinephrine group and for 18 patients the dopamine group.

#The number of patients who treated the volume of infusion within 30 minutes of arrival at emergency department was divided by the number of patients who the pathophysiology of shock was “volume”.

ACS, acute coronary syndrome; SBP, systolic blood pressure; ED, emergency department; IQR, interquartile range; HR, heart rate; RR, respiratory rate; BT, body temperature; LVEF, left ventricular ejection fraction.

Supplementary Table 5. Patient characteristics in Group G3b

| **Characters** | **Norepinephrine group (n=29)** | **Dopamine group (n=43)** | **P value** |
| --- | --- | --- | --- |
| **Age-yr median (IQR)** | **79 (69– 84)** | **78 (72- 82)** | **0.739** |
| **Male sex - no. (%)** | **15 (51.7)** | **26 (60.5)** | **0.463** |
| **The pathophysiology of shock – no (%)** |  |  | **0.625** |
| **Pump** | **15 (51.7)** | **22 (51.2)** |  |
| **Volume** | **5 (17.2)** | **11 (25.6)** |  |
| **Rate** | **9 (31.0)** | **10 (23.3)** |  |
| **Cardiogenic source – no. (%)** |  |  | **0.237** |
| **ACS** | **16 (55.2)** | **14 (32.6)** |  |
| **Arrhythmia** | **1 (3.4)** | **7 (16.3)** |  |
| **Aortic disease** | **3 (10.3)** | **13 (30.2)** |  |
| **The others** | **9 (31.0)** | **9 (20.9)** |  |
| **SBP on ED arrival-mmHg median (IQR)** | **80 (56- 90)** | **76 (60- 85)** | **0.571** |
| **HR on ED arrival- beats/min median (IQR)** | **76 (55- 94)** | **90 (40- 106)** | **0.612** |
| **RR on ED arrival- per min median (IQR)** | **20 (16- 25)** | **20 (14- 26)** | **0.531** |
| **BT on ED – degree centigrade median (IQR)** | **36.0 (35.0- 36.0)** | **36.0 (35.0- 36.0)** | **0.230** |
| **Arterial pH on ED arrival* median (IQR)** | **7.33 (7.24- 7.41)** | **7.29 (7.15- 7.38)** | **0.172** |
| **Arterial Lactate on ED arrival –mmol/l** median (IQR)** | **2.85 (1.03- 6.98)** | **6.70 (3.10- 8.00)** | **0.326** |
| **eGFR at ED arrival median (IQR)** | **40.8 (35.2 – 43.7)** | **38.6 (34.0 – 42.0)** | **0.109** |
| **LVEF on ED arrival median (IQR)** | **50.0 (40.5- 58.8)** | **53.0 (40.0- 67.0)** | **0.662** |
| **Heart failure, -no. (%)** | **14 (48.3)** | **25 (58.1)** | **0.410** |
| **Out-of–hospital cardiac arrest before hospital arrival, – no. (%)** | **6 (20.7)** | **11 (25.6)** | **0.632** |
| **Mechanical ventilation - no. (%)** | **20 (69.0)** | **23 (53.5)** | **0.189** |
| **Continuous hemodiafiltration - no. (%)** | **0 (0)** | **2 (4.7)** | **0.239** |
| **Patients treated anti-arrhythmic agents within 12 hours of arrival at ED- no. (%)** | **0 (0)** | **3 (7.0)** | **0.350** |
| **The volume of infusion within 30 minutes of arrival at ED – no. (%)^#^** |  |  | **0.541** |
| **≤ 500ml** | **1 / 5 (20.0)** | **1 / 11 (9.1)** |  |
| **> 500ml** | **4 / 5 (80.0)** | **10 / 11 (90.9)** |  |

*The arterial ph was recorded for 22 patients in the norepinephrine group and for 34 patients in the dopamine group.

**The arterial lactate was recorded for 18 patients in the norepinephrine group and for 25 patients the dopamine group.

#The number of patients who treated the volume of infusion within 30 minutes of arrival at emergency department was divided by the number of patients who the pathophysiology of shock was “volume”.

ACS, acute coronary syndrome; SBP, systolic blood pressure; ED, emergency department; IQR, interquartile range; HR, heart rate; RR, respiratory rate; BT, body temperature; LVEF, left ventricular ejection fraction.

Supplementary Table 6. Patient characteristics in Group G4/5

| **Characters** | **Norepinephrine group (n=27)** | **Dopamine group (n=35)** | **P value** |
| --- | --- | --- | --- |
| **Age-yr median (IQR)** | **77 (70– 83)** | **82 (70- 87)** | **0.280** |
| **Male sex - no. (%)** | **16 (59.3)** | **18 (61.4)** | **0.539** |
| **The** **pathophysiology of shock – no (%)** |  |  | **0.186** |
| **Pump** | **15 (55.6)** | **15 (42.9)** |  |
| **Volume** | **7 (25.9)** | **6 (17.1)** |  |
| **Rate** | **5 (18.5)** | **14 (40.0)** |  |
| **Cardiogenic source – no. (%)** |  |  | **0.536** |
| **ACS** | **5 (18.5)** | **8 (22.9)** |  |
| **Arrhythmia** | **5 (18.5)** | **11 (31.4)** |  |
| **Aortic disease** | **5 (18.5)** | **8 (22.9)** |  |
| **The others** | **12 (44.4)** | **8 (22.9)** |  |
| **SBP on ED arrival-mmHg median (IQR)** | **77 (55- 87)** | **70 (47- 83)** | **0.336** |
| **HR on ED arrival- beats/min median (IQR)** | **80 (36- 112)** | **51 (24- 104)** | **0.456** |
| **RR on ED arrival- per min median (IQR)** | **24 (13- 29)** | **20 (12- 26)** | **0.466** |
| **BT on ED – degree centigrade median (IQR)** | **36.0 (34.8- 37.0)** | **36.0 (34.8- 36.0)** | **0.302** |
| **Arterial pH on ED arrival* median (IQR)** | **7.29 (7.12- 7.38)** | **7.29 (7.00- 7.39)** | **0.877** |
| **Arterial Lactate on ED arrival –mmol/l** median (IQR)** | **3.10 (0.85- 10.7)** | **4.60 (2.90- 6.70)** | **0.631** |
| **eGFR at ED arrival median (IQR)** | **16.6 (10.5 – 22.8)** | **19.1 (15.5 – 25.2)** | **0.209** |
| **LVEF on ED arrival median (IQR)** | **33.5 (28.5- 60.0)** | **48.0 (30.0- 67.0)** | **0.225** |
| **Heart failure, -no. (%)** | **21 (77.8)** | **27 (77.1)** | **0.953** |
| **Out-of–hospital cardiac arrest before hospital arrival, – no. (%)** | **6 (22.2)** | **11 (31.4)** | **0.420** |
| **Mechanical ventilation - no. (%)** | **15 (55.6)** | **25 (71.4)** | **0.195** |
| **Continuous hemodiafiltration - no. (%)** | **2 (7.4)** | **2 (5.7)** | **0.788** |
| **Patients treated anti-arrhythmic agents within 12 hours of arrival at ED- no. (%)** | **5 (18.5)** | **2 (5.7)** | **0.002** |
| **The volume of infusion within 30 minutes of arrival at ED – no. (%)^#^** |  |  | **0.725** |
| **≤ 500ml** | **3 / 7 (42.9)** | **2 / 6 (33.3)** |  |
| **> 500ml** | **4 / 7 (57.1)** | **4 / 6 (66.7)** |  |

*The arterial ph was recorded for 21 patients in the norepinephrine group and for 32 patients in the dopamine group.

**The arterial lactate was recorded for 13 patients in the norepinephrine group and for 19 patients the dopamine group.

#The number of patients who treated the volume of infusion within 30 minutes of arrival at emergency department was divided by the number of patients who the pathophysiology of shock was “volume”.

ACS, acute coronary syndrome; SBP, systolic blood pressure; ED, emergency department; IQR, interquartile range; HR, heart rate; RR, respiratory rate; BT, body temperature; LVEF, left ventricular ejection fraction.
